# Eliminating oncogenic RAS: back to the future at the drawing board

**DOI:** 10.1042/BST20221343

**Published:** 2023-01-23

**Authors:** Candy Laura Steffen, Pelin Kaya, Elisabeth Schaffner-Reckinger, Daniel Abankwa

**Affiliations:** Cancer Cell Biology and Drug Discovery Group, Department of Life Sciences and Medicine, University of Luxembourg, L-4362 Esch-sur-Alzette, Luxembourg

**Keywords:** cancer, drug development, RAS

## Abstract

RAS drug development has made enormous strides in the past ten years, with the first direct KRAS inhibitor being approved in 2021. However, despite the clinical success of covalent KRAS-G12C inhibitors, we are immediately confronted with resistances as commonly found with targeted drugs. Previously believed to be undruggable due to its lack of obvious druggable pockets, a couple of new approaches to hit this much feared oncogene have now been carved out. We here concisely review these approaches to directly target four druggable sites of RAS from various angles. Our analysis focuses on the lessons learnt during the development of allele-specific covalent and non-covalent RAS inhibitors, the potential of macromolecular binders to facilitate the discovery and validation of targetable sites on RAS and finally an outlook on a future that may engage more small molecule binders to become drugs. We foresee that the latter could happen mainly in two ways: First, non-covalent small molecule inhibitors may be derived from the development of covalent binders. Second, reversible small molecule binders could be utilized for novel targeting modalities, such as degraders of RAS. Provided that degraders eliminate RAS by recruiting differentially expressed E3-ligases, this approach could enable unprecedented tissue- or developmental stage-specific destruction of RAS with potential advantages for on-target toxicity. We conclude that novel creative ideas continue to be important to exterminate RAS in cancer and other RAS pathway-driven diseases, such as RASopathies.

## Introduction

The small GTPase RAS operates as a switchable recruitment site of downstream effectors to the membrane. Thus GTP-binding triggers multiple intracellular signalling pathways, notably the MAPK pathway, which drives proliferation and differentiation [1]. This central position to orchestrate hallmarks of life may explain why RAS is so frequently exploited in cancer, where the three RAS genes, *KRAS*, *NRAS* and *HRAS* combined are mutated in 19% of cancer patients [2]. Mutations typically occur in hotspot codons 12, 13 or 61, which essentially keep RAS GTP-bound and thus constitutively active.

In 2021 the first direct RAS inhibitor, sotorasib (AMG 510), was approved after a 40 year long quest to inhibit this major oncogene. Impressive initial clinical data with a median overall survival of 12.5 months in smoking-associated KRAS-G12C mutant NSCLC patients supported this effort [3]. A number of other G12C-specific inhibitors are currently being evaluated in patients, including adagrasib (MRTX849), which is the second G12C-inhibitor to enter clinical assessment [4,5]. However, the application of these inhibitors is limited to KRAS-G12C mutant tumours, such as found in 14% of NSCLC patients, and <5% in colorectal and pancreatic cancers. Moreover, emerging resistances have stunted overall patient response and the initially high expectations. Resistance mechanisms include additional oncogenic KRAS mutations in codons 12, 13 or 61 that are not susceptible to G12C-inhibitors [6,7].

Nonetheless, the first direct RAS inhibitors are a tremendous first milestone that demarcate the extraordinary achievements in RAS drug development during the past decade. They impressively demonstrate what happens, if specifically the oncogenic version of a major cancer driver is drug-targeted. Yet they also clarify that even with exquisite (covalent) on-target specificity, side effects cannot be ruled out [8]. Most importantly, these inhibitors provide unequivocal proof of KRAS as a cancer drug target in humans.

The KRAS-G12C inhibitor development story is testimony to not take no for an answer, and pursue the targeting of cancer drivers, even if they were considered undruggable. This justifies and encourages novel drug development efforts against RAS. We will here review, which approaches are on the drawing boards of researchers and give an outlook on potential future developments.

## The development of allele-specific and pan-RAS inhibitors for clinical applications

**Figure 1.**
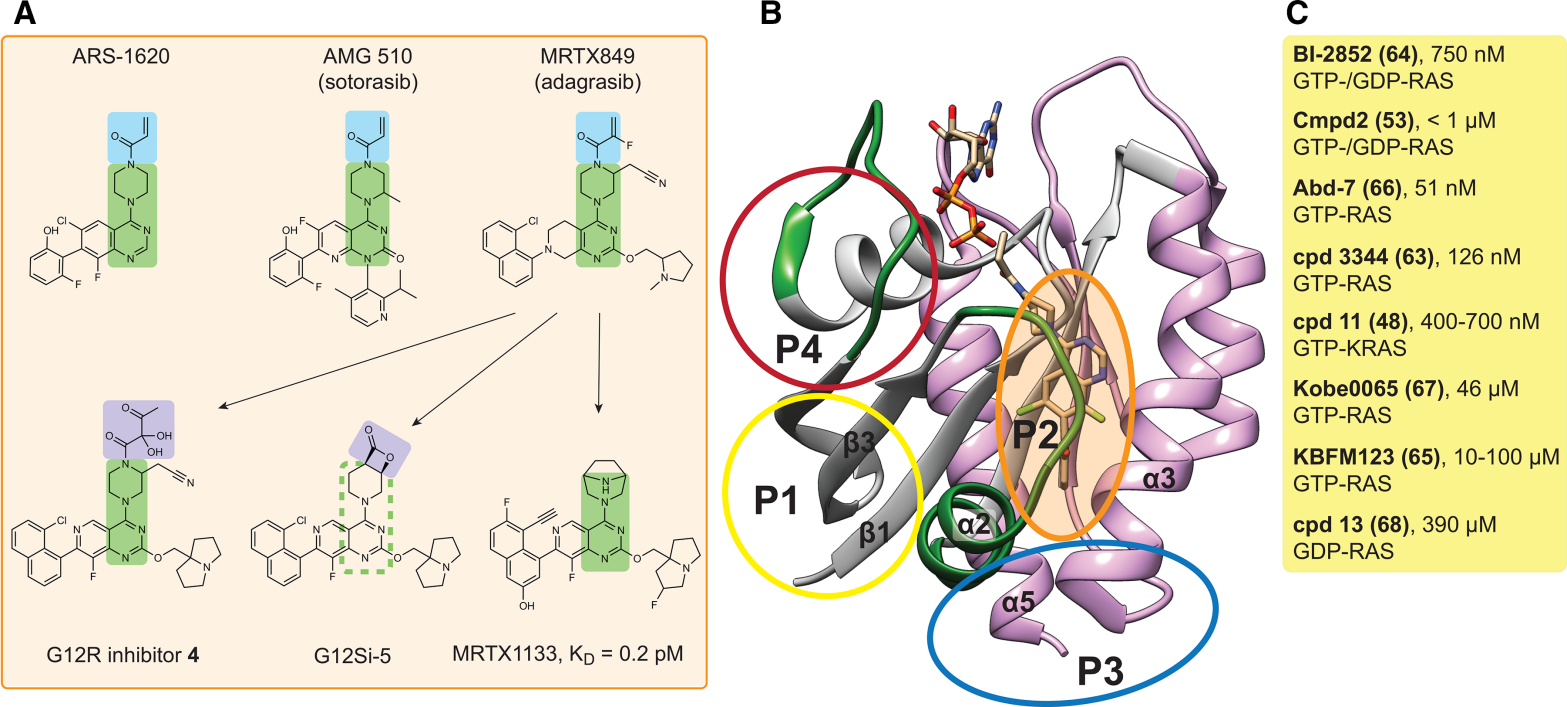
Overview of small molecule inhibitors targeting RAS. (**A**) Selected SII-P small molecule inhibitors based on the 4-piperazin-1-yl-pyrimidine scaffold (green highlights). The common acrylamide warhead of KRAS-G12C inhibitors (top row) is highlighted in blue. Adagrasib served as a starting point for additional inhibitors (arrows), including covalent G12R- and G12S-inhibitors, with an α,β-diketoamide warhead or a strained β-lactone electrophile, respectively (purple). Note that the exact stereochemistry of displayed inhibitors has been largely omitted. (**B**) Crystal structure of GDP-KRAS-G12C in complex with ARS-1620 (PDB ID 5V9U). The RAS structure can be divided into the N-terminal effector lobe (grey), with the switch I and switch II regions labelled in green, and the allosteric lobe (pink). The allosteric binding sites P1–4 are indicated with circles. (**C**) Current experimental small molecule inhibitors (here those with an affinity <500 µM) target predominantly P1. The RAS affinity and selectivity is indicated for each compound (cpd). References are in brackets after the names [48,53,63–68]. The full list of small molecule inhibitors is contained in Supplementary File S1.

Crystal structures of RAS show that GTP-binding induces conformational changes in two regions of RAS, called switch I and switch II, without revealing targetable pockets on RAS [1]. However, seminal work from the Shokat group published in 2013 identified the cryptic allosteric switch II-pocket (SII-P), which manifests only upon binding of KRAS-G12C inhibitors [9]. Their first proof-of-concept inhibitor introduced the acrylamide warhead for covalent engagement of the nucleophilic cysteine on position 12, thus creating a paradigm that has until today been widely utilized (Figure 1). Since then, essentially every major pharma company has developed KRAS-G12C inhibitors and we refer to recent reviews for details on their pre-/clinical progress [5,10].

The common chemical theme of these compounds in addition to their identical warhead is the 4-piperazin-1-yl-pyrimidine scaffold core that was essentially introduced with ARS-1620 [11]. Intriguingly, with the development of the scaffold of adagrasib a significant non-covalent binding to wild-type KRAS and to a number of KRAS mutants that carry hotspot mutations on codons 12, 13 and 61 was achieved [12]. In line with this, the adagrasib scaffold served as a starting point for the development of the first covalent inhibitors of KRAS-G12S and KRAS-G12R in the GDP-bound OFF-state [13,14]. These carry instead of the acrylamide warhead, a strained β-lactone electrophile in the case of the G12S-inhibitor, while an α,β-diketoamide warhead was used in the G12R-inhibitor (Figure 1A). All of these SII-P targeting compounds lock KRAS in an inactive conformation by distorting switch I and switch II, thus typically blocking access of RAS activating GEFs, such as SOS, and of RAS effectors, notably RAF [9,11–14]. In agreement with the reuse of the pharmacologically validated adagrasib scaffold, inhibitors are furthermore active in cells, to suppress MAPK signalling and selectively the growth of cancer cells carrying the targeted mutation.

One initially puzzling finding was that all of these covalent inhibitors rely on the GDP-bound, inactive KRAS. However, oncogenic KRAS mutants are generally approximated to be constitutively GTP-bound and ON. While it is commonly assumed that the GTPase activating protein (GAP) neurofibromin (NF1) turns RAS OFF, the heterotrimeric G protein-associated GAP RGS3 was identified as the enzyme that sufficiently inactivates all major oncogenic KRAS alleles [15]. Consequently, ablation of RGS3 severely decreased the anti-tumorigenic effect of adagrasib in a mouse xenograft model. This can be explained by the distinct catalytic mechanisms of NF1 and RGS3. NF1 provides a catalytic arginine (the Arg-finger) to speed-up GTP hydrolysis of RAS, a mechanism that is crucially inhibited by oncogenic hotspot mutants of RAS [16]. In contrast, RGS3 is from a different family of GAPs, which likely bind RAS also involving its switch regions, but employ asparagine as catalytic residue [17,18].

It is astonishing, but not the first time in RAS/ MAPK biology that such a fundamental biological mechanism was only discovered after the first RAS inhibitors entered the clinic. Both failure of farnesyl transferase inhibitors and paradoxical RAF activation were only fully recognized at the clinical stage [10]. The RGS3-catalyzed hydrolysis of RAS furthermore begs the question, in which biological context then is the NF1-associated GAP-activity required, given that all hotspot mutants of RAS evade it.

The OFF-state dependency of SII-P inhibitors is also liable to major resistance mechanisms, which increase the ON-state, such as mutational activation of EGFR or up-regulation of other receptor tyrosine kinases [5]. Additional resistance mechanisms after sotorasib treatment include mutations that disrupt binding of the inhibitors to the SII-P, most notably Y96D, which also blocks access of adagrasib [6,19]. *In vitro* studies furthermore forecast evasive mutations, which increase GTP-levels of KRAS, such as Y40A, N116H and A146V [20]. Xenograft data furthermore suggest that MAPK pathway reactivation occurs sooner or later in particular by the emergence of clones with other oncogenic KRAS alleles or overactivation of other RAS isoforms, including MRAS [7].

Some of these resistance issues can be overcome by inhibiting the ON-state of KRAS. The adagrasib-derived non-covalent inhibitor MRTX-EX185 demonstrates this potential even for a SII-P binder [12]. The non-covalent inhibitor MRTX1133 exploited this further and introduced sub-picomolar targeting of the most common KRAS mutation, KRAS-G12D, with potent inhibition of signalling and xenograft growth [21].

Another embodiment is seen in a completely different RAS inhibition approach that is being evaluated in clinical trials. A whole panel of allele-specific and pan-RAS inhibitors has been commercially developed, which tie together KRAS in the ON-state and the ubiquitous and abundant chaperone protein cyclophilin A [22]. These ‘molecular glue’ compounds lead to an inhibitory tri-complex formation that sterically blocks RAS interactions and thus downstream signalling. Molecular glues are small molecules, which link two proteins in a non-native complex to inhibit or modify at least one of the binding partners [23]. The interesting potential of this approach is demonstrated by the covalent KRAS-G12C inhibitor RM-018, which can overcome the Y96D-dependent resistance encountered with sotorasib and adagrasib [19]. In addition to KRAS-G12C, the tri-complex approach has been utilized to covalently target KRAS-G12D, KRAS-G13C and multiple RAS alleles non-covalently, as recently reviewed elsewhere [5].

## The exploration of novel binding sites and inhibition principles of RAS using macromolecular binders

In the commercial tri-complex approach, binding to the part of RAS that engages effectors is obstructed. This first half of the RAS protein (residues 1–85) is therefore also referred to as effector lobe, while the second half of the G-domain (residues 86–166) is called the allosteric lobe. The effector lobe makes major contacts not only with effectors, but all other major regulators of RAS, such as GEFs and GAPs.

Therefore, high affinity macromolecular binders raised against the effector lobe can potently inhibit RAS signalling. In addition to classical antibodies (∼150 kDa) and Fab-fragments (∼50 kDa), much smaller specific binders can be raised by directed evolution *in vitro*, such as designed ankyrin repeat proteins (DARPins; ∼20 kDa), Affimers (∼12 kDa), which are based on the artificial phytocystatin-derived scaffold called Adhiron, and monobodies (∼10 kDa), which originate from an artificial fibronectin type III domain [24–26]. Such binders exhibit typically affinities in the nanomolar range and encode high binding specificities to a small contact area. The small contact site can be exploited for pharmacophore based computational or *in vitro* competitive screening for small molecule functional analogues.

**Figure 2.**
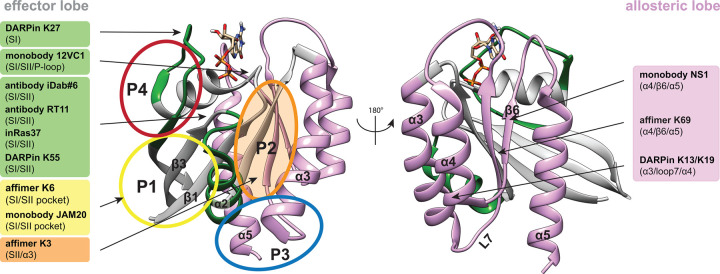
Overview of macromolecular RAS binders. Crystal structure of GDP-KRAS (PDB ID 4OBE). Effector and allosteric lobes, as well as allosteric binding sites are indicated as in Figure 1. The names of macromolecular RAS binders are highlighted in the same colour as their binding sites, with more detailed binding site information given in brackets.

Obvious targets on the effector lobe are the switch regions, for which both GTP-specific binders (antibodies iDab#6, RT11, inRas37, monobody 12VC1, DARPin K55) [27–30], as well as GDP-specific binders (monobody JAM20, DARPin K27) have been identified [27,31] (Figure 2). Accordingly, these reagents typically repress RAS/ effector-binding and RAS-activation, respectively, and several were shown to block RAS-mutant cancer cell growth *in vitro* and in murine tumour models.

The truly exciting potential of these artificial binders lies in their ability to discover novel binding sites on RAS, which is notoriously binding cavity free. In support of this potential, affimer K3 was found to bind at the same site of KRAS, where current covalent G12C-inhibitors are lodging. Similarly, another affimer K6 binds to a pocket in between the switch I and switch II regions, a site that is also targeted by inhibitors DCAI and BI-2852 (Figure 1 and Supplementary File S1) [32–34].

Several other macromolecular binders engage with RAS on the allosteric lobe, hence in a nucleotide-independent manner. Complexation creates significant sterical bulk around RAS, which plausibly impacts on higher complex formation, such as transient dimers and nanoclustering. Nanoclusters are proteo-lipid complexes containing transient di-/trimeric RAS assemblies, which act as membrane recruitment sites of RAF-effectors and are therefore necessary for MAPK signalling [35]. In addition, the conformational mobility of RAS at the membrane impacts on MAPK signalling [35–38]. Given that a bulky binder would most probably restrain such conformational motions it is plausible to assume that they also affect associated RAS activities.

The monobody NS1 binds to HRAS and KRAS, but not NRAS, at an epitope comprising helices α4 and α5 [39]. These make up the most common interface that is assumed to partake in RAS self-organization into nanoclusters on the plasma membrane [40]. This interface was also recognized by the affimer K69 [32]. In contrast, the DARPins K13 and K19 bind to helices α3 and α4, which have also been suggested as interface for transient RAS dimers at the membrane [40]. While such macromolecular binders are *per se* not pharmacologically tractable for an intracellular target such as RAS, they nevertheless provide crucial proof-of-concept data for the target site in cellular and *in vivo* models.

Moreover, they can be further functionalized to enable new modes of action. By genetically fusing E3-ligase subunits such as von Hippel-Lindau (VHL) tumour suppressor to the monobodies NS1 and 12VC1 or the DARPin K19, RAS degrader constructs were generated [30,41,42]. In general degraders realized potent RAS signalling suppression and anti-proliferative activities, and in the case of the 12VC1 were also more potent than the competitively binding monobody alone [30]. Given that these degraders emulate the proteolysis targeting chimera (PROTAC) mode of action, which will be discussed in the next chapter, they may be useful to forecast the potential of analogous PROTACs [43].

On the pathway to develop smaller RAS binders, peptides are a natural intermediate. A number of peptides or peptidomimetics that target the GTP-KRAS effector lobe typically with nanomolar affinity and compete with effector binding and downstream signalling of RAS have been developed. These peptides have a median size of ∼14 residues, can be either linear or cyclic, and contain non-natural amino acids or other chemical modifications (i.e. peptidomimetics) (Table 1). Cyclic peptides are entropically advantageous and are more resistant against exopeptidases [44]. So far, none of these peptides have been harnessed for degrader development.

**Table 1:** Overview of RAS binding peptides

Name (PDB ID)	RAS specificity	*K*_D_ (nM)	Site on RAS	Properties	Ref.
**Linear**					
RBDv1, RBDv12	GTP-RAS	3.35 2.52	P4	14 aa, inhibits RAS signalling, reduces cancer cell growth	[69]
SAH-SOS1	GDP-/GTP-RAS	106–175	near P4	16 aa, blocks nucleotide exchange, reduces cancer cell growth	[70]
225-11 (5WPL)	GTP-RAS	3.3	P4	32 aa, blocks effector interaction	[71]
R11.1.6 (5UFQ)	RAS-G12D	4	switch II	61 aa, blocks effector interaction, inhibits RAS signalling	[72]
**Cyclic**					
Cyclorasin 9A5	GTP-RAS	440	near P4	11 aa, blocks effector interaction, inhibits RAS signalling	[73]
Cyclorasin B4-27	GTP-RAS	21	near P4	16 aa, blocks effector interaction (cellular BRET-assay)	[74]
KRpep-2d (5XCO)	KRAS-G12D	51	P2	19 aa, inhibits RAS signalling, reduces cancer cell growth	[75–77]
KS-58	KRAS-G12D	22	P2	11 aa, inhibits RAS signalling, reduces cancer cell growth *in vivo*	[78,79]
KD2 (6WGN)	GTP-KRAS-G12D	none	near P2	15 aa, blocks effector interaction	[80]

Peptide and peptidomimetic RAS binders and their properties. The PDB ID is given if the complex with RAS was determined.

## What is the future of RAS inhibition? From small molecule binders to PROTAC-degraders

RAS is a small mono-domain protein with a shallow surface that has been considered undruggable due to the lack of obvious binding pockets. The nucleotide binding site remains problematic as a target, due to the high cellular GTP concentration in combination with the picomolar affinity of the guanine nucleotides to RAS [5]. However, computational approaches led by the Gorfe group, have identified already in 2011, hence well before the discovery of first covalent inhibitors, altogether four low affinity (sub-/millimolar) allosteric sites on RAS named P1 to P4 that have all been experimentally validated [45–47]. P1 and P4 are situated in the effector lobe, P3 in the allosteric lobe and P2 in between both lobes (Figure 1B).

The hydrophobic pocket P1 is located between switch II and β-strands 1–3 and is partially closed in crystal structures of GDP-RAS [48]. It essentially corresponds to the switch I/switch II region that is targeted by several experimental ON- and OFF-state binders (Figure 1C and Supplementary File S1). P2 is at the interface of helix α2 with helix α3. This cryptic hydrophobic pocket is currently the most successfully targeted site, as it harbours the covalent OFF-state inhibitors targeting G12C, G12S, G12R and non-covalent inhibitors targeting G12D (Figure 1A). The polar P3 site is located between helix α5 and loop 7 and is accessible in both GTP- and GDP-states of KRAS, but less in the other RAS isoforms [46]. However, currently few binders target this site, such as metal cyclens and KAL-21404358 [49,50]. P4 is also polar and situated behind switch I and possesses andrographolide derivatives as the most interesting ligands currently [51]. It thus appears that the number of targetable sites on RAS is limited.

By combining computational and experimental approaches several small molecules have been identified that bind primarily to P1 and P2 (Figure 1C and Supplementary File S1). These ligands cover a broad range of affinities from milli- to nanomolar, typically lack RAS isoform selectivity and can disrupt binding of RAS interaction partners, such as RAF, and suppress MAPK signalling or cell viability. Only for compound 11 was KRAS-selective on-target binding demonstrated *in vitro* [48]. Therefore, cellular effects of low affinity compounds have to be taken with caution, as at the early stages of compound discovery off-target effects will contribute to these readouts.

With the exception of the covalent and non-covalent SII-P binding inhibitors, none of the small molecule binders has advanced toward clinical development. This may suggest that before a non-covalent inhibitor (such as MRTX1133) can flourish, a covalent counterpart that is anchored at the desired site may be advantageous during compound development [9].

Given their size, small molecules are less likely to block protein–protein interfaces such as needed to inhibit RAS nanoclustering. However, membrane-bound RAS also undergoes potentially RAS isoform specific conformational changes that impact on its nanoclustering [36,37]. Interestingly, some very rare cancer-associated and RASopathy mutations seem to affect nanoclustering by perturbing conformational dynamics of RAS [38,52]. A similar conformational shift may therefore also be achievable by small molecules, which was indeed demonstrated by the Ikura group. They showed that Cmpd2 stabilizes a non-productive conformation of KRAS at the membrane, by binding in between the membrane and the P1 site [53]. Another intriguing concept originated from the serendipitous discovery of a RAS-dimer stabilizer BI-2852, which was developed as RAS switch I/switch II pocket binder [33,54]. This nanomolar ligand illustrates the potential to modulate RAS oligomerization, specifically by locking it in a non-productive dimer.

As compared with competitive inhibitors, PROTACs instruct protein degradation by recruiting the ubiquitin-proteasome system to the target protein [55]. They can therefore bind outside of an active or allosteric site of a protein and after degradation abrogate any scaffolding functions of the target. This is enabled by their hybrid structure, which contains one binder (the warhead) for the target protein that is tethered via a linker to a moiety that recruits an E3-ligase, most commonly VHL and cereblon. The latter was enabled by the finding that immunomodulatory thalidomide derivatives alone work as ‘molecular glues’ that stick cereblon to IKAROS-family transcription factors and thus instruct their degradation [55].

Both concepts, molecular glues and PROTACs are thus not only historically related but bear similar capabilities, as both types of inhibitors can be potentially reused after reversible binding to and degradation of the target protein. Of note, molecular glues may also act by incapacitating a protein in a non-functional complex, such as illustrated by the tri-complex approach described earlier. Given that PROTACs follow an apparent ‘plug-and-play’ design, where the E3-ligase recruiting moiety can be utilized in several molecules, this approach currently predominates [55]. However, significant optimization for linker length and pharmacological properties of the relatively large molecules still requires substantial developmental efforts [56].

Current RAS-targeting PROTACs (XY-4-88, LC-2, KP-14) all build on the covalent G12C-inhibitors and as such cannot benefit from PROTAC degrader recycling, as these inhibitors are consumed due to the covalent cysteine engagement (Supplementary File S1) [57–59]. An interesting advancement in this regard is the development of reversible covalent inhibitor YF135, which employs a cyanoacrylamide for cysteine linkage [60]. Side-by-side comparison with the RAS-binding warhead alone furthermore demonstrates a 30-fold higher activity of the PROTAC. It remains to be seen, how and whether any of the exploratory RAS-ligands (Figure 1C and Supplementary File S1) can be converted into PROTACs. Given the distinct spatio-temporal expression of some E3-ligases in tissues and inside of cells, PROTACs may provide a more controlled drug action, which could reduce toxicity and new treatment mechanisms [61,62].

**Figure 3.**
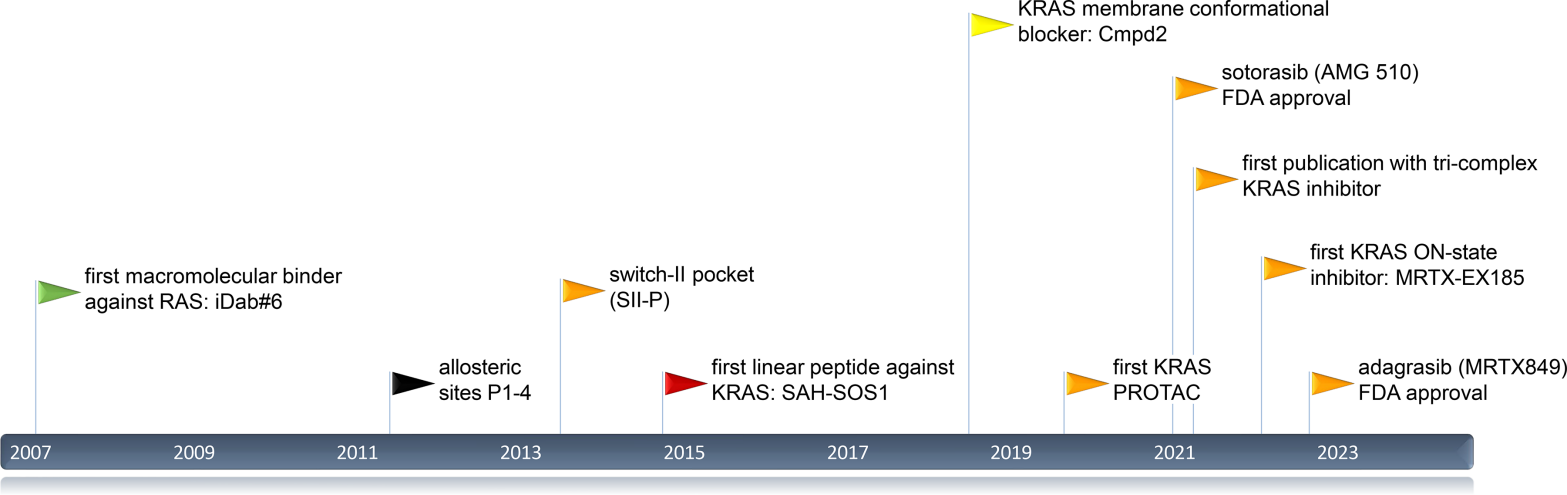
Timeline of notable RAS drug development events since 2007. Arrowheads mark publications of binders and sites with colours corresponding to those used for binding sites in Figures 1 and 2.

RAS drug development is in full motion since 2007 (Figure 3) and it can be hoped that novel creative ideas will continue to provide new RAS drugs for cancer therapy or other RAS-associated diseases, such as RASopathies.

## Perspectives

- KRAS is the most frequently mutated oncogene and a major driver of cancer (stemness), which has finally become a clinically validated drug-target, thanks to KRAS-G12C targeting sotorasib and adagrasib. However, the performance of these compounds in the clinic warrants continuing efforts in RAS pathway drug development and further research to understand the essence of RAS in cancer.
- At least four targetable allosteric pockets and four surface areas on RAS have been identified and validated by the discovery of macromolecular-, peptidic- and small molecule-binders. These block upstream processes of RAS signalling, such as effector binding and nanoclustering.
- PROTAC degraders of RAS may offer new ways to inhibit RAS in a spatio-temporally (tissue type, differentiation stage, cell-cycle stage) more defined manner, with potential benefits for on-target toxicity. However, the viability of this approach awaits evaluation in the clinic.

## Abbreviations

EGFR: Epidermal growth factor receptor
GEF: Guanine nucleotide exchange factor
MAPK: Mitogen-activated protein kinases
NSCLC: Non-small cell lung cancer
RGS3: Regulator of G-protein signalling 3
SOS: Son of sevenless guanine nucleotide exchange factor
